# Non-invasive, Focused Ultrasound-Facilitated Gene Delivery for Optogenetics

**DOI:** 10.1038/srep39955

**Published:** 2017-01-06

**Authors:** Shutao Wang, Tara Kugelman, Amanda Buch, Mathieu Herman, Yang Han, Maria Eleni Karakatsani, S. Abid Hussaini, Karen Duff, Elisa E. Konofagou

**Affiliations:** 1 Department of Biomedical Engineering, Columbia University, New York, USA; 2 Department of Pathology and Cell Biology, Columbia University, New York, USA

## Abstract

Optogenetics, a widely used technique in neuroscience research, is often limited by its invasive nature of application. Here, we present a noninvasive, ultrasound-based technique to introduce optogenetic channels into the brain by temporarily opening the blood-brain barrier (BBB). We demonstrate the efficiency of the method developed and evaluate the bioactivity of the non-invasively introduced channelrhodopsin channels by performing stimulation in freely behaving mice.

Optogenetics, the introduction of light-activated protein channels into mammalian cells, has revolutionized the field of neuroscience by enabling researchers to manipulate neuronal activities in a controlled manner. Discovered in algae, the light-sensitive proteins such as Channelrhodopsin-2 (ChR2) are generally introduced to the targeted mammalian neurons via viral gene delivery approaches^1^. Due to the existence of the BBB, the only feasible route for viral vector delivery is through direct brain infusion^2^. The invasive infusion procedures inevitably cause damage to the brain regions of interest and the infused viral vectors often undergo backflow along the inserted cannula leading to insufficient gene expression^3^. An alternative approach is to use transgenic mice that express light-activated protein channels^4^. Albeit it being non-invasive, this method suffers from full body genome alteration and high cost of animal model development. Therefore, a more suitable technique to introduce light-activated protein to mammalian neurons would preferably be non-invasive, flexible to a diverse group of proteins and relatively low cost.

With recent advancement in transcranial focused ultrasound (FUS) technology, non-invasive and targeted BBB opening became possible in several animal models^5^,^6^. This technique involves a systemic injection of a mixture composed of ultrasound contrast agents (lipid-based microbubbles) and molecules to be delivered. The emitted ultrasonic waves propagate through the skull and cause the microbubbles to cavitate (to oscillate) within the capillaries in the targeted brain region^7^,^8^. It was reported that this interaction transiently loosens the tight junctions between endothelial cells^9^. As a result, the BBB is temporarily opened and the molecules of interest diffuse into the brain parenchyma according to their concentration gradient^10^. By taking the advantage of the non-invasive nature of the FUS technique, we report here the design and implementation of a FUS-facilitated gene delivery for optogenetic applications. Viral vectors encoding various light-activated protein channels delivered via this approach allow for an entirely non-invasive neural stimulation procedure *in vivo*.

## Results

**Figure 1:**
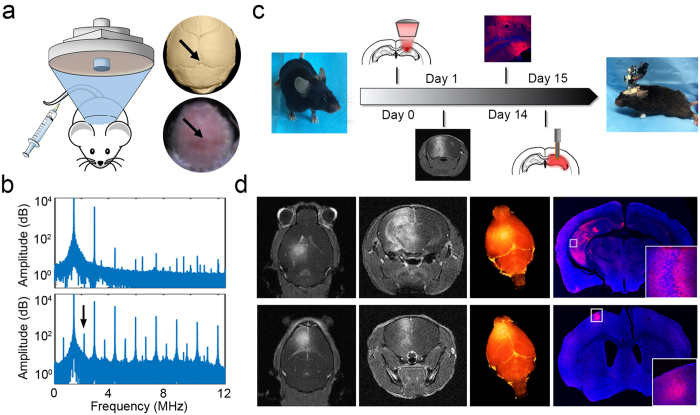
FUS-facilitated viral delivery of ChR2 protein and safety evaluation. (**a**) Experimental schematic and the targeted brain region was precisely located by identifying the lambda suture through shaved scalp. (**b**) The timeline for our technique. Subjects first received systematic injection of microbubbles and viral vectors, followed by FUS sonication. The BBB opening was confirmed via MRI and the animals were allowed to survive for 2 weeks. The bioactivity of the expressed ChR2 was evaluated by performing optical stimulation at the targeted area. (**c**) Signals collected from a passive cavitation detector (PCD): top panel shows the spectra of pre-microbubble injection signals, while ultra-harmonic signals (e.g. black arrow) indicate ongoing cavitation events. (**d**) BBB opening confirmation with contrast-enhanced MRI in hippocampus (top panel) and cortex (bottom panel) and the corresponding ChR2 expression (red, last column).

One of the major advantages of transcranial FUS is its accurate region selectivity. Depending on the region of interest, precise targeting can be achieved by identifying the lambda suture through depilated scalp (Fig. 1a). Using this approach, we first carried out FUS sonications in wild-type mice to transiently open the BBB with a 1.5-MHz ultrasound transducer. Immediately prior to each sonication, a mixture of lipid-shelled microbubbles and adeno-associated virus (AAV) serotype 9 encoded for ChR2 was intravenously injected via the tail vein. The sonication procedure was monitored using a passive cavitation detector (PCD), where the signals emitted from the microbubble oscillation were collected and analyzed. The top panel in Fig. 1b is an example of the PCD spectra prior to administering microbubbles. In contrast, the appearances of ultra-harmonic signals (e.g. arrow pointing spike in Fig. 1b bottom panel) and higher harmonic signals signify the interactions between the ultrasonic wave and the administered microbubbles. The success of BBB opening was confirmed via contrast-enhanced, T1-weighted magnetic resonance imaging (MRI), where bright pixels indicate regions diffused by the MR contrast agent. We then allowed the animals to survive for two weeks so that sufficient ChR2 protein channels were expressed in the targeted brain regions. Since one of our goals was to assess the comparison of the non-invasive FUS-based technique to the commonly used direct infusion approach, we implanted an electrode apparatus for optical stimulation and signal recording at the end of the two-week period. A full timeline of our experimental design is shown in Fig. 1c. We demonstrated the versatility of the FUS-facilitated gene delivery by targeting different structures in the brain. As shown in Fig. 1d, the hippocampus (top panel) and cortex (bottom panel) were targeted in different subjects. The BBB-opened regions are depicted as bright pixels in the horizontal and coronal MR images. The reconstructed three-dimensional images present a clear view of the volume of the BBB opening. Brain regions at various depths can be selected by adapting the ultrasound focus.

**Figure 2:**
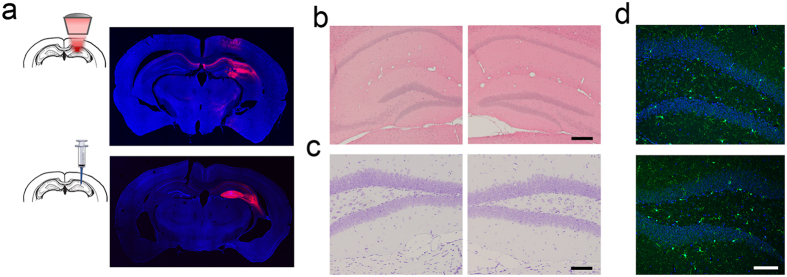
(**a**) Comparison of ChR2 expression using FUS-facilitated viral delivery (top) and the direct infusion technique (bottom). (**b-c**) Hematoxylin and Eosin (H&E) staining (**b**) and Nissl staining (**c**) were used to evaluate potential structural damage (N = 3, right column is FUS sonicated side). No damage was found in any subjects. (**d**) Microglia staining revealed no notable inflammatory response on the FUS sonicated side (bottom) compared to the contralateral side (top). Scale bar in (**b**) indicates 200 μm, while in (**c,d**) represent 100 μm.

We compared the efficiency of FUS-facilitated AAV delivery with direct infusion. As shown in Fig. 2a, abundant viral transduction was observed in both the FUS-facilitated delivery and direct infusion. To ensure that the FUS-facilitated gene delivery technique does not cause any physiological damage to the brain, we performed histolopathological analysis on the animals that received unilateral sonication. Based on the Hematoxylin and Eosin (H&E) and Nissl staining results (shown in Fig. 2b and c), no structural damage was observed in either the sonicated side (right column) or the contralateral side (left column). Furthermore, microglia staining (Fig. 2d) revealed no notable inflammatory response caused by FUS.

**Figure 3:**
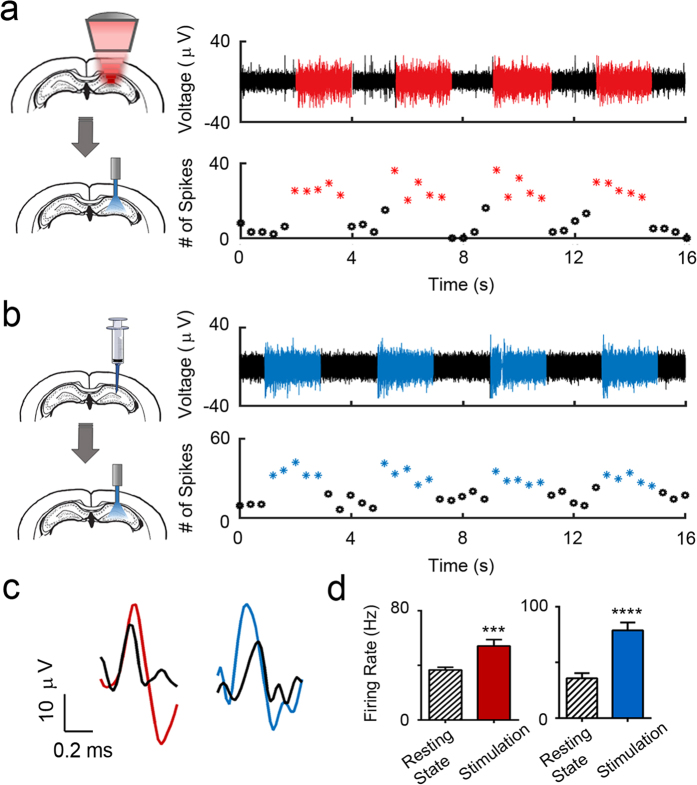
The bioactivity of FUS-facilitated viral delivery was tested via optical stimulation. (**a**) Four 2 s blue light (470 nm) pulses were given to elicit neuronal response in freely behaving mice. FUS-facilitated viral delivery was carried out in these mice (N = 3) targeting hippocampus. Sorted spike signals (top) and the corresponding number of spikes (bottom, binned into 0.4 s segments) revealed elevated neuronal activity during stimulation (red). (**b**) Four 2-s pulses were applied to mice (N = 3) that received direct infusion of viral vectors. An increased neuronal activity was observed during stimulation, which is similar to that of FUS-facilitated viral delivery group. (**c**) Examples of individual spike signals during stimulation from FUS-facilitated delivery group (red) and direct infusion group (blue). The baseline level spike for each group is indicated in black. (**d**) The firing rate was calculated for each group and significant increases (p < 0.001) in firing rate were observed during stimulation.

We then investigated the bioactivity of the delivered viral vectors and the expressed ChR2 proteins by stimulating the hippocampus in freely behaving mice with the blue light (470 nm) at 30 Hz. Four 2-s pulses were applied and the evoked neuronal activity was recorded as described in a previously published study^11^. The recorded signals were spike-sorted and the total number of spikes were binned into 0.4 s segments. Figure 3a is an example of optical stimulation at the hippocampus that was carried out in a mouse receiving FUS-facilitated viral delivery. Each red segment corresponds to a 2-s stimulation ‘on’ time, while the black segments represent stimulation ‘off’ periods (resting state). As evidenced by the bottom panel, elevated number of spikes were observed during each stimulation pulse indicating the proper functionality of the ChR2 protein channels. For the purpose of comparison, we performed commonly used direct infusion procedures in a separate group of mice. Figure 3b demonstrates recorded spike signals and corresponding number of spikes. Similar to the FUS-facilitated delivery group, an increase in the amplitude and number of spikes per second was observed during the 2-s stimulation on periods (as indicated in blue). A control study where no FUS was applied (AAV injected intravenously) was performed with similar stimulations (Supplementary Fig. 1). No change in signal amplitude nor the number of spikes were observed in these subjects. Examples of individual spike signals during stimulation for the FUS-facilitated delivery group (red) and direct infusion group (blue) are shown in Fig. 3c. Quantitatively, we calculated the neuronal firing rate for each group (n = 4) based on the number of spikes (Fig. 3d). Compared to the baseline level, significant increases of firing rate were observed in both the FUS-facilitated delivery group (p < 0.001) and direct infusion group (p < 0.001).

**Figure 4:**
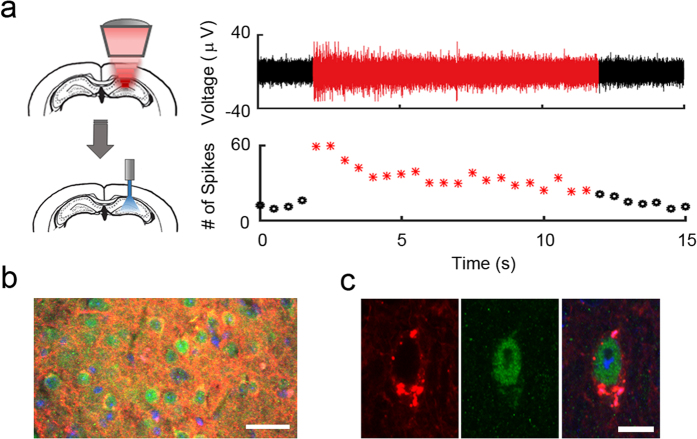
(**a**) Ten-second long pulses were also applied (red). An increased spike amplitude and number of spikes were observed at the onset of stimulation, which was followed by a gradual decrease to baseline level. (**b**) Neuronal activity (ChR2 is red) was revealed by labeling c-Fos proteins with fluorescent markers (green). (**c**) An example of a ChR2-expressing neuron that was activated by optical stimulation. Scale bars represent 20 μm and 5 μm in (**b**,**c**), respectively.

Additionally, a 10-s continuous stimulation pulse was given and the corresponding spike signal is shown in Fig. 4a. In this case, the total number of spikes were binned into 0.5-s segments. At the onset of the long pulse stimulation, the number of spikes rose to 118 spikes/sec and was then followed by a gradual decrease to the baseline level. This observation can be attributed to the depletion of the ion concentration gradient upon prolonged stimulation. Finally, an enhanced level of c-Fos (marker for neuronal activity) was detected via immunofluorescence staining at the regions that express ChR2 and received optical stimulation. As shown in Fig. 4b, mcherry (red, co-encoded in the viral genome) was used to mark ChR2 proteins, while c-Fos was labelled in green. The high magnification images (Fig. 4c) are an example of the activation of a ChR2-expressing neuron indicating the successfully elicited neuronal activity.

## Discussion

We have introduced and implemented a FUS-based, non-invasive viral delivery technique for optogenetic stimulation. We report comparable volumes of transduction and bioactivity between the FUS method and the commonly used direct infusion technique. The difference lies in that direct infusion results in a more confined volume of gene expression while the FUS-based technique produced a more diffuse-transduction. If desired, a single FUS sonication may generate a substantially large axial volume of BBB opening in the coronal plane due to the associated FUS beam shape. The volume of opening, which also determines the volume of viral transduction, can be altered through appropriate selection of the ultrasound parameters. For instance, in Fig. 1d, the focus was placed 2 mm below the skull when targeting hippocampus and almost the entire hippocampus was transduced (indicated by the pink mCherry protein). In contrast, when cortex was the target, the ultrasound focus was placed 0.5 mm below the skull. Given the intravenous nature of the viral administration in the FUS-based method, approximately 20 times more viral vectors were used (~4 × 10^11^ GC/animal). Although the absolute number of genome copies was not quantified in each group, the overall transduction volumes were comparable between the two groups, which assumes a 5% of the viral vectors crossing the opened BBB. It is worth noting that the intravenously injected viral vectors circulate systemically. As a result, we found viral transduction in the liver, kidney and heart tissue by directly imaging mcherry expression (data not shown). However, unintended viral transduction can be minimized by carefully selecting appropriate promotors, such as synapsin for neuron-specific transduction^12^.

Our technique might be preferable because of its versatility and entirely non-invasive nature, providing an ideal condition to study neuronal activities *in vivo*. It might therefore prove particularly useful for researchers who are interested in using long-wavelength light to transcranially stimulate intact brain regions. Furthermore, our technique can be combined with sonogenetics^13^ where the expression of mechano-sensitive ion channels is enhanced to achieve fully ultrasound-controlled neuromodulation. The electrode implantation phase can be eliminated to achieve complete non-invasive transcranial stimulation when appropriate optical wavelength is used, such as the red light-driven neural inhibitor reported by Chuong *et al*.^14^ and red-shifted variant of channelrhodopsin developed by Lin *et al*.^15^. A limitation of this technique is the potential barrier of commercialization and the requirement of basic experience with therapeutic ultrasound technology. The implementation of the protocol requires a separate set of focused ultrasound equipment and careful selection of parameters. The viral vector itself does not need to be altered for the proposed non-invasive technique, though further dosage optimization should be performed to obtain most efficient transduction.

## Methods

### Microbubble and viral vector

The lipid-shelled microbubbles were manufactured in-house following a previously published protocol^16^. Briefly, the 1,2-distearoyl-sn-glycero-3-phosphocholine (DSPC) and polyethylene Glycol 2000 (PEG2000) were mixed at a 9:1 ratio. Two milligrams of the mixture was dissolved in a 2 ml solution consisted of filtered PBS/glycerol (10% volume)/propylene glycol (10% volume) using a sonicator (Model 1510, Branson Ultrasonics, Danbury, CT, USA) and stored in a 5 ml vial. The remainder of the vial was filled with decafluorobutane (C_4_F_10_) gas. The vial was then activated via mechanical agitation using VialMix^TM^ (Lantheus Medical Imaging, N. Billerica, MA) shaker for a pre-set time of 45 s. The formed microbubbles were analyzed with a Coulter Counter Multisizer (Beckman Coulter Inc., Fullerton, CA). The distribution of these microbubbles is shown in Supplementary Fig. 1 and the mean diameter was measured to be 0.9 μm. The viral vectors used in this study was AAV9-mcherry-ChR2 with a titer of 2.48 × 10^13^ GC/ml (Penn Vector Core, Philadelphia, PA). The vectors were diluted with phosphate buffered saline (PBS) at a 1:6 ratio before administration.

### FUS sonication

A single element FUS transducer (focal length: 60 mm and radius: 30 mm, Imasonic, France) was used for all sonications in this study. The transducer has a center frequency of 1.5 MHz and a −6 dB focus of 7.5 × 1 × 1 mm^3^ as determined by a needle hydrophone (Supplementary Fig. 2). To monitor cavitation events, a pulse-echo transducer (radius: 11.2 mm, focal length: 60 mm, and center frequency: 10 MHz, Olympus NDT, Waltham, MA) was confocally aligned with the FUS transducer. The pulse-echo transducer was driven by a pulser-receiver (Olympus, Waltham, MA), which was connected to a digitizer (Gage Applied technologies, Inc., Lachine, QC, Canada) for data acquisition. The pulse-receiver was operated on a “receive mode” and served as an amplifier during PCD acquisition. The transducer setup was then mounted onto a three-dimensional positioning system (Velmex Inc., Lachine, QC, Canada) for accurate targeting.

Targeting of specific brain regions was achieved by first visualizing the lambda suture through the shaved scalp which was described in greater details elsewhere^17^. Briefly, a small water tank with an ultrasound transparent opening was placed on top of the shaved scalp and a metallic cross was used to locate the lambda suture. C-mode ultrasound scans were then carried out to identify the position of the cross and therefore the lambda suture. The FUS transducer was then moved via the positioner to the following coordinates: AP +2.6 mm, ML −2 mm and DV +2.5 mm for hippocampus targeting; AP +6.3 mm, ML −1 mm and DV +0.5 mm for motor cortex targeting. For each target, a single sonication was performed with the following acoustic parameters: a free field (i.e. in water) peak rarefactional pressure (PRP) of 0.74 MPa, pulse repetition frequency (PRF) 5 Hz, pulse length 10 ms, and a total duration of 120 s. The collected cavitation signal was analyzed using a custom written program (MATLAB R2011a, MathWorks, Inc., Natick, MA). The appearance of ultraharmonics and broadband noise was deemed the signature of cavitation events. Immediately prior to the sonication, a mixture of 100 μl diluted AAV vectors and 5 μl microbubbles were injected intravenously via the tail vein. All experimental procedures involving animals were approved by the Columbia University Institutional Animal Care and Use Committee and in accordance to the Office of Laboratory Animal Welfare and the Association for Assessment and Accreditation of Laboratory Care regulations.

### MRI

The BBB opening was confirmed with T1-weighted contrast enhanced MR imaging (9.4 T, Bruker Medical, Boston, MA). Upon the completion of the sonication, a bolus of 0.15 ml of gadodiamide (GD-DTPA) (Omniscan^®^, GE Healthcare, Princeton, NJ) was administered intraperitoneally to each mouse. Approximately 50 min post GD-DTPA injection, the mice were placed in a birdcage coil (diameter 3 cm) and imaging was performed with a T1-weighted 2D FLASH sequence (TR/TE 230/3.3 ms, flip angle 70°, 20 slices, 10 averages, bandwidth 100 kHz, matrix size 256 × 256, resolution 100 μm × 100 μm × 400 μm).

### Surgery and optical stimulation

For mice that received direct virus infusion, the following procedures were carried out. Mice were anesthetized with a mixture of oxygen and 1–3% isoflurane (SurgiVet, Smiths Medical PM, Inc., WI) and placed prone with its head immobilized by a stereotaxic apparatus (David Kopf Instruments, Tujunga, CA). The head was shaved and an incision was made to expose the skull. The hippocampus was located using the following coordinates centered at the bregma: AP −2.7 mm, ML −2 mm and DV +1.5 mm. A 34 G needle (Hamilton, Reno, NV) was used to infuse a total volume of 0.8 μl AAV vectors at approximately 0.003 μl/s. Upon completion, the needle was left in place for an additional 5 min to minimize back flow. Mice that received either FUS-facilitated viral delivery (N = 3) or direct infusion (N = 3) were allowed to survive two weeks before the implantation surgery. An optical fiber (Thorlabs Inc. Newton, NJ) and microdrive (Axona Ltd. UK) assembly was implanted to the targeted brain region for optical stimulation and neural activity recording. About 3–4 jeweler’s screws were inserted into the skull to support the implants. An additional screw connected with wire was also inserted into the skull which served as a reference electrode. Dental cement was spread across the exposed skull to secure the optical fiber and the microdrive assembly. The mice were allowed one day to recover from the surgery before undergoing optical stimulation. During each session, the optical fiber was connected to a LED light source and was stimulated with four 2-second pulses and one 10-second pulse of 470 nm (blue) light at 30 Hz frequency. The mouse implanted with electrodes was plugged to the electrophysiology setup (Axona) during each stimulation and neuronal activity was recorded as described previously^11^. The recorded signals were analyzed and visualized with a custom-written program (MATLAB R2011a, MathWorks, Inc., Natick, MA). To quantify the number of spikes, a threshold was set at three standard deviations above the mean of the resting state signals. Peaks above the threshold were deemed to be a spike and was binned into either 0.4 s or 0.5 s segments. The firing rate was quantified as the total number of spikes per second.

### Histology and imaging

One hour post optical stimulations (for maximal c-Fos expression), mice were sacrificed and transcardially perfused with 30 mL PBS and followed by 60 mL 4% paraformaldehyde. The skull was removed upon sacrifice and the brain was soaked in paraformaldehyde overnight. The brain tissue was then cryo-protected with 30% sucrose solution for 48 hours and frozen on dry ice. Brain samples were sectioned coronally at 40 μm and mounted with ProLong^®^ Gold mounting solution with DAPI (ThermoFisher, Waltham, MA) to examine the distribution of ChR2 (mcherry). Neuronal activation was visualized via immunochemistry staining for c-Fos proteins. Brain sections were incubated with primary anti-c-Fos antibody (ab209794, Abcam, Cambridge, MA) at 1:100 dilution, and secondary antibody (R37116, ThermoFisher, Waltham, MA) at 1:250 dilution. All fluorescence images were taken with a Nikon confocal microscope (Nikon Instruments Inc., Melville, NY). A separate group of mice (N = 3) was used to evaluate the safety aspects of the FUS sonication. One day post the sonication, these mice were sacrificed as previously described and soaked in paraformaldehyde for two days. The brains were then paraffin fixed and serially sectioned at 7 μm. The structural integrity of the brain was examined via Hematoxylin and Eosin (H&E) staining and Nissl staining. Potential inflammatory response was evaluated by staining for microglia. The paraffin sections first underwent antigen retrieval process followed by incubation in primary anti-Iba-1 antibody (1:200, ab5076, Abcam, Cambridge, MA) and secondary donkey anti-goat Alexa 488 (1:200, A-11055, Invitrogen, Carlsbad, CA).

### Statistical analysis

All statistical analyses were performed using GraphPad software (GraphPad Software, Inc., La Jolla, CA, USA). Unpaired two-tailed Student’s t-test was used to compare the volume of macromolecule delivery and a p-value < 0.05 was considered statistically significant.

## Additional Information

**How to cite this article**: Wang, S. *et al*. Non-invasive, Focused Ultrasound-Facilitated Gene Delivery for Optogenetics. *Sci. Rep.* **7**, 39955; doi: 10.1038/srep39955 (2017).

**Publisher's note:** Springer Nature remains neutral with regard to jurisdictional claims in published maps and institutional affiliations.

## Supplementary Material

Supplementary Figure 1
