# Supplementary material for: Non-invasive, Focused Ultrasound-Facilitated Gene Delivery for Optogenetics
Source: Sci Rep. 2017 Jan 6;7:39955. doi: 10.1038/srep39955 (PMC5216389; doi:10.1038/srep39955)
Supplement: Supplementary Figure 1 [file srep39955-s1.pdf]

Non-invasive, Focused Ultrasound-Facilitated Gene Delivery for Optogenetics

Shutao Wang[1], Tara Kugelman[1], Amanda Buch[1], Mathieu Herman[2], Yang Han[1], Maria Eleni Karakatsani[1], S. Abid Hussaini[2], Karen Duff[2] & Elisa E. Konofagou[1]*

Supplementary Figure1

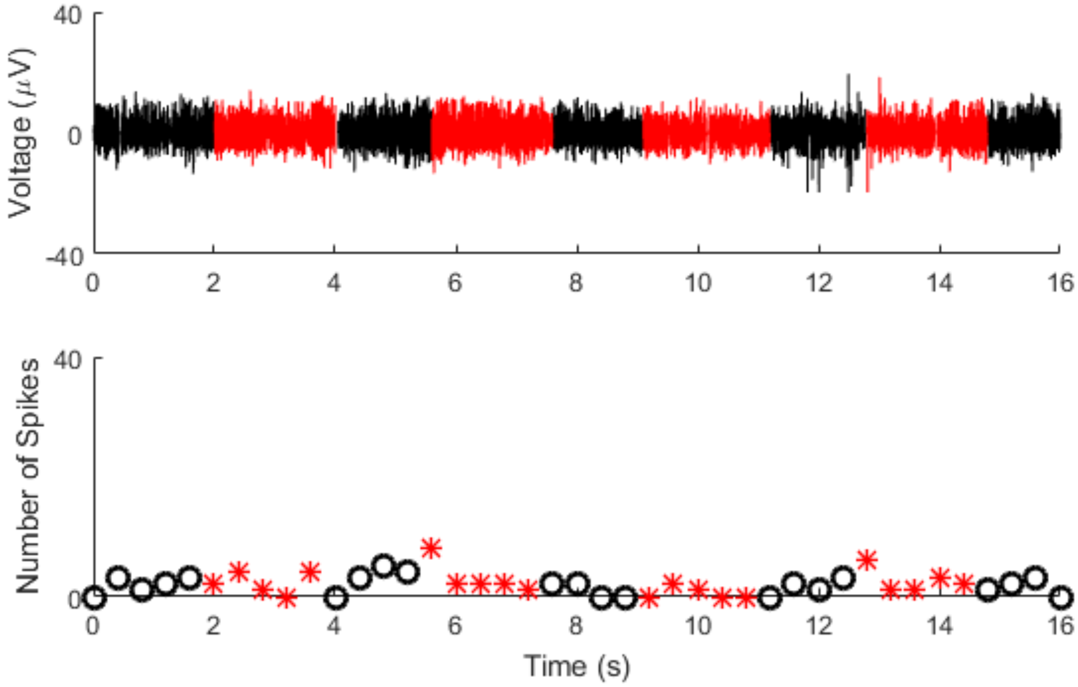

Four 2-s pulses were applied to mice (N = 3) that received I.V. injection of AAV and no FUS. No change was observed when stimulation was turned on.
